# Mate Choice Drives Evolutionary Stability in a Hybrid Complex

**DOI:** 10.1371/journal.pone.0132760

**Published:** 2015-07-16

**Authors:** Miguel Morgado-Santos, Henrique Miguel Pereira, Luís Vicente, Maria João Collares-Pereira

**Affiliations:** 1 CE3C: Centre for Ecology, Evolution and Environmental Changes, Faculdade de Ciências da Universidade de Lisboa, 1749–016, Lisboa, Portugal; 2 CESAM-Lisboa: Centro de Estudos do Ambiente e do Mar–Departamento de Biologia Animal, Faculdade de Ciências da Universidade de Lisboa, 1749–016, Lisboa, Portugal; 3 iDiv: German Centre for Integrative Biodiversity Research, Halle-Jena-Leipzig, Germany; 4 CFCUL: Centro de Filosofia das Ciências da Universidade de Lisboa, Faculdade de Ciências da Universidade de Lisboa, 1749–016, Lisboa, Portugal; University of Massachusetts, UNITED STATES

## Abstract

Previous studies have shown that assortative mating acts as a driver of speciation by countering hybridization between two populations of the same species (pre-zygotic isolation) or through mate choice among the hybrids (hybrid speciation). In both speciation types, assortative mating promotes speciation over a transient hybridization stage. We studied mate choice in a hybrid vertebrate complex, the allopolyploid fish *Squalius alburnoides*. This complex is composed by several genomotypes connected by an intricate reproductive dynamics. We developed a model that predicts the hybrid complex can persist when females exhibit particular mate choice patterns. Our model is able to reproduce the diversity of population dynamic outcomes found in nature, namely the dominance of the triploids and the dominance of the tetraploids, depending on female mate choice patterns and frequency of the parental species. Experimental mate choice trials showed that females exhibit the preferences predicted by the model. Thus, despite the known role of assortative mating in driving speciation, our findings suggest that certain mate choice patterns can instead hinder speciation and support the persistence of hybrids over time without speciation or extinction.

## Introduction

Many studies have shown that assortative mating acts as a driver of speciation [1–3], especially through the reinforcement of pre-zygotic isolation [4–8]. However, the relationship between assortative mating and hybrid speciation is still not well understood. Hybrid speciation occurs when two species reproduce to form hybrid organisms which over time evolve into a new species, with or without genome multiplication (allopolyploid and homoploid hybrid speciation, respectively) [9, 10]. By recreating the original hybridization events through experimental crosses between the parental species, some empirical studies suggested a key role for mate choice in driving homoploid hybrid speciation [11–13]. However, the role of mate choice in allopolyploid speciation remains unknown. Here, we studied mate choice in a well-established allopolyploid organism which may be on the verge of hybrid speciation [14].

**Fig 1:**
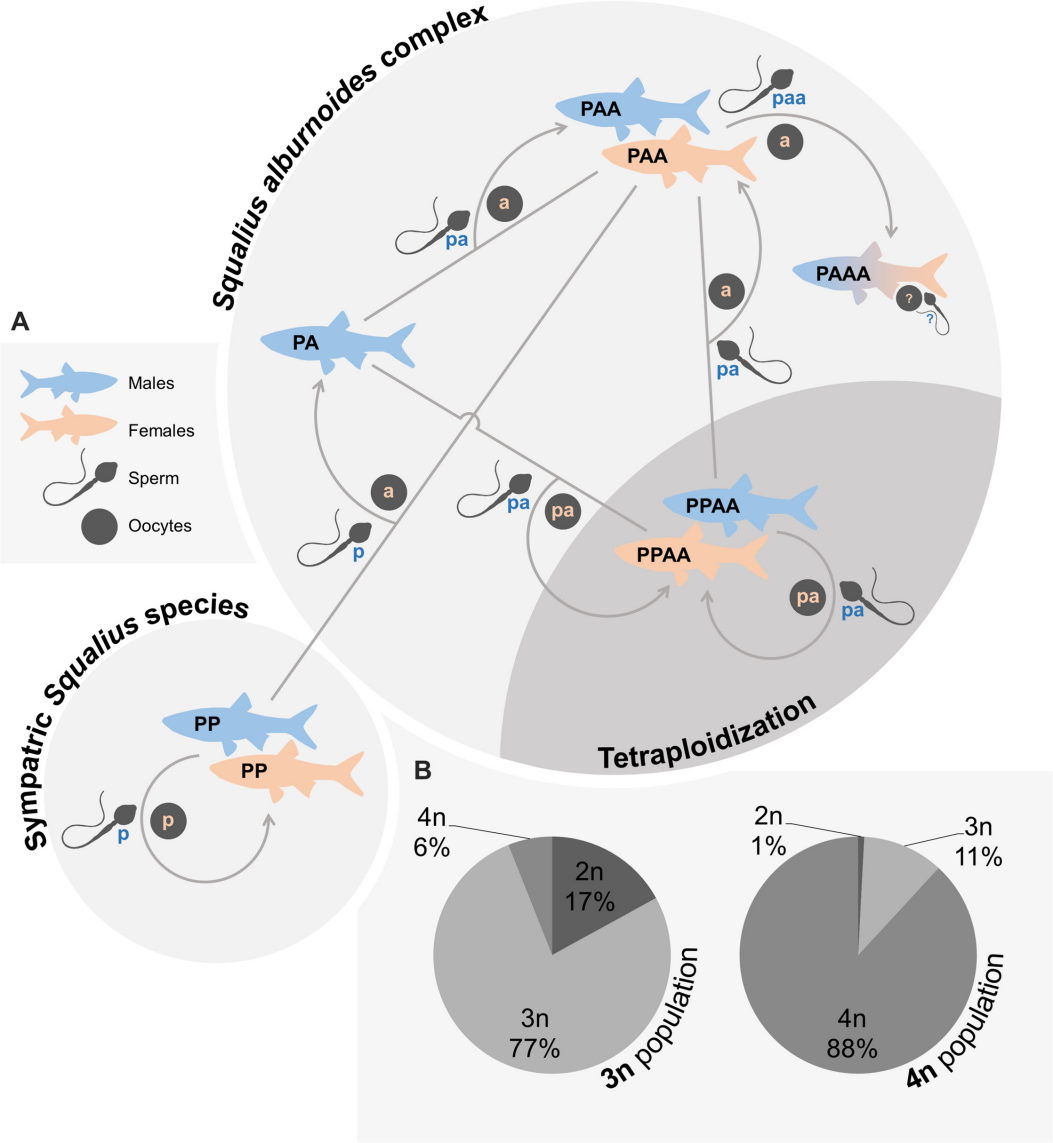
Reproductive dynamics and example genomotype compositions of the *S*. *alburnoides* complex. **A.** Simplified diagram of *S*. *alburnoides* reproductive dynamics in a triploid-dominated (light grey area) and in a tetraploid-dominated (dark grey area) populations. See Introduction for more details about the reproductive modes of each genomotype. The genome nomenclature used was based on central and southern populations where the bisexual *Squalius* non-hybrid species is *S*. *pyrenaicus* (PP). Other non-hybrid *Squalius* species are also sympatric with *S*. *alburnoides* in other geographic regions (*S*. *carolitertii*, CC genome, in northern populations and *S*. *aradensis*, QQ genome, in an isolated southwestern population), but their involvement in the reproductive dynamics of the complex is identical to the one shown. Very little is known about the extremely rare PAAA genomotype and, thus, their sex ratio and reproductive modes remain unknown. For other reproductive dynamics found in natural populations see the review by [16]. **B.** Examples of triploid-dominated and tetraploid-dominated populations. The shown ploidy levels only refer to the *S*. *alburnoides* genomotypes, being the proportions of the diploid *Squalius* non-hybrid species not represented on the charts.

*Squalius alburnoides* is an Iberian freshwater fish originated by the hybridization of females of the still sympatric *Squalius pyrenaicus* (PP genome, P oocytes) with males from an extinct species related to the extant *Anaecypris hispanica* (AA genome, A sperm) [15]. These intergeneric crosses produced fertile hybrids (PA genome) with clonal gametogenesis (PA gametes). In turn, crosses between these allodiploids and backcrosses with the parental species originated a successful hybrid complex that includes fertile males and females with distinct ploidies (2n = 50, 3n = 75 and 4n = 100) and different combinations of the parental genomes (genomotypes) (reviewed in [16]). This diversity of fertile genomotypes enables a multiplicity of crosses, with females being able to mate with several distinct male genomotypes (Fig 1A).

*Squalius alburnoides* natural populations vary in their composition of genomotypes. Two distinct population types with utterly distinct reproductive dynamics may be defined, namely triploid-dominated and tetraploid-dominated populations (Fig 1B). The overall sex-ratio of triploid-dominated populations is highly female-biased, with males only representing around 15% of the allotriploid genomotype (PAA) that dominate in such populations. PAA females may breed with any of the male genomotypes available in the population, namely allodiploid (PA), allotriploid (PAA) and balanced tetraploid (PPAA) males, but also males from the sympatric *S*. *pyrenaicus* non-hybrid species (PP) (Fig 1A). PAA females reproduce by meiotic hybridogenesis, a reproductive mode in which the heterospecific genome (P) is discarded and the remaining homologous genomes (AA) undergo meiosis, producing haploid oocytes (A) [16]. Consequently, these females may generate three types of offspring: a) PAA offspring from crosses with PA or PPAA males (which produce PA sperm through clonal gametogenesis and meiosis, respectively), restocking the triploid genomotype in the population; b) PA offspring from crosses with the sympatric *S*. *pyrenaicus* non-hybrid species (which produces, bisexually, P sperm through meiosis); and c) PAAA offspring from crosses with PAA males (which produce PAA sperm through clonal spermatogenesis) (reviewed in [15, 16]). In triploid-dominated populations, most genomotypes are interdependent, meaning their production depends exclusively on crosses involving other genomotypes (Fig 1A). Thus, triploid-dominated populations rely on the maintenance of a high variability of genomotypes in order to persist over time.

The same requirement does not apply to the tetraploid-dominated populations because they are mainly composed by the only *S*. *alburnoides* self-sustainable genomotype (PPAA). The PPAA genomotype has a balanced sex ratio, with males and females producing allodiploid (PA) sperm and oocytes through meiosis [16]. Thus, the offspring produced by crosses between PPAA males and females is also PPAA, not requiring the involvement of any other genomotype and also of the sympatric *Squalius* non-hybrid species. This independency not only allows for a much simpler reproductive dynamics in tetraploid-dominated populations (Fig 1A), but also potentiates hybrid speciation through assortative mating [14]. That is, if PPAA females show a stronger preference for PPAA males over other male genomotypes (assortative mating), this would favor the evolution of a new independent species, an evolutionary pathway not available in triploid-dominated populations due to their obligatory genomotype interdependency. In fact, classic assortative mating is not possible to occur among the PAA genomotype because crosses between PAA males and females do not father PAA offspring, meaning PAA females have to mate disassortatively in order to produce offspring of their own type. Moreover, the offspring produced when PAA females mate assortatively (PAAA genomotype) is extremely rare in natural populations, which suggests that assortative mating is unlikely to be occurring among the PAA genomotype. Thus, triploid-dominated populations only succeed if PAA females have a less strict mate choice pattern, allowing them to mate with distinct male forms and, therefore, maintain genomotype variability. Indeed, other studies have suggested that mate choice plasticity allows the maintenance of polymorphisms in natural populations of several species [17–21].

The two *S*. *alburnoides* population types are not evenly found in the wild. Whereas triploid-dominated populations abound across *S*. *alburnoides* distribution range, only two tetraploid-dominated populations have been found so far [14]. This pattern suggests that the reproductive strategies ruling each population type may not be equally successful. The flexible mate choice patterns occurring among the triploid genomotype seem to overrule the effect of the assortative mating occurring among the tetraploid one, thus preventing tetraploidization and maintaining most populations triploid-dominated (i.e. in their hybrid state). If so, mate choice may be hindering hybrid speciation in *S*. *alburnoides* complex.

In order to test this hypothesis, we simulated the theoretical effect of a wide range of mate preferences and genomotype frequencies in shaping the composition of natural populations over time. This theoretical approach was complemented by experimental trials, aimed at studying mate choice in *S*. *alburnoides* PAA females, allowing them to choose among the available male genomotypes. The results obtained were assessed in order to evaluate how the observed mate choice patterns of the most common and abundant female genomotype influence the genomotype composition of the offspring produced and whether such mate tendencies route or counter tetraploidization and, consequently, hybrid speciation.

## Materials and Methods

### Ethics Statement

Fish captures were carried out with the permission of Instituto da Conservação da Natureza e das Florestas (permit numbers 140/2012/CAPT and 239/2013/CAPT). Although the taxa studied are threatened, the population chosen for sampling (Ocreza River, Tagus drainage) was not imperiled and the sample size was chosen to avoid depletion of the natural stock. Fishes were handled following recommended ethical guidelines [22]. Electrofishing was performed in low duration pulses to avoid killing juveniles (300 V, 2–4 A) and the transport to the laboratory was made in appropriate aerated containers. The portion of fin used for genomotype assessment was minimum and the removal was performed in a peripheral area of the fin in order to guarantee a fast regrowth of the tissue and minimize fish discomfort after awakening from the anesthesia (0.1 g/L MS-222, 0.2 g/L NaHCO_3_). The study was not carried out on private land and all specimens were returned alive to the collecting site after the experiments.

### Theoretical Modeling

We formulated a theoretical model for the dynamics of the genomotype frequencies in a *S*. *alburnoides* population, using female mating preference and *S*. *pyrenaicus* frequency as model parameters.

**Fig 2:**
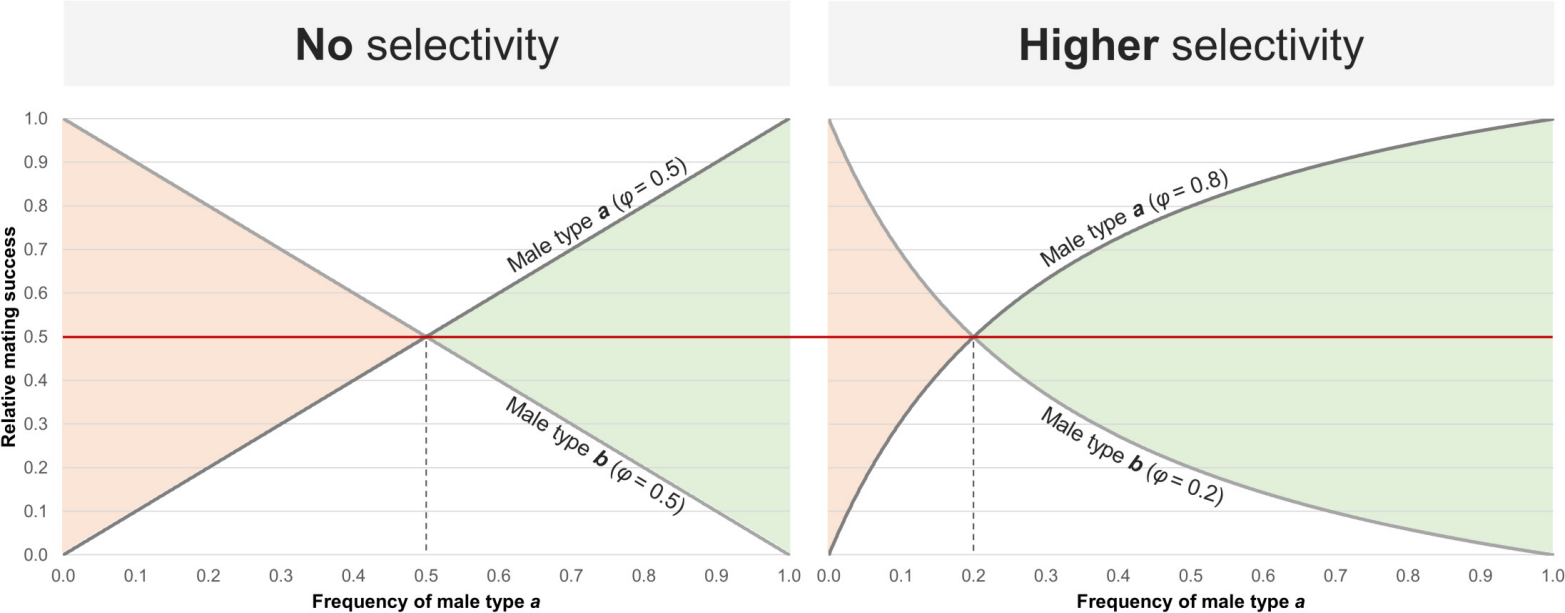
Relative mating success of two theoretical male types as a function of their frequency and female preference (*φ*). The intersection between both lines bounds two areas: the red area, in which male type *b* has a higher relative mating success, and the green area, in which male type *a* has a higher relative mating success. If female preference is similar towards both male types (*φ* = 0.5; i.e. females choosing male type *a* or male type *b* for mating is equally probable), the male relative mating success depends exclusively on their frequency in the population (assuming that the effect of all other synergistic factors affecting male mating success are similar for both male types). However, if females show a higher preference for a particular male type (e.g. *φ* = 0.8 for male type *a*), the frequency-based functions of the male relative mating success deviate, increasing the green area and decreasing the red area, that is, increasing the relative mating success for male type *a* and decreasing it for male type *b*.

The frequency of each male and female genomotype in a population determines the encounter probability of two particular genomotypes. This encounter probability would be directly proportional to mating success if there were no other factors, such as differential mate choice, affecting or biasing cross occurrence. In the former case, the probability of a female mating with a male of a given type would be a linear function of the frequency of those males. Adding the effect of female mate preference leads to a non-linear response and an increase of the mating probability with the favored males (Fig 2). Note that, because *S*. *alburnoides* is a multiple spawner, females can breed with distinct male genomotypes and produce distinct offspring in a single reproductive season. Considering that, at time *t*, a particular male genomotype (*M*_*j*_) occurs with a frequency *f*_*Mj*_ in the population, and a particular female genomotype (*F*_*i*_), occurring with *f*_*Fi*_ frequency, has *φ*_*Fi→Mj*_ preference for that male genomotype, then the probability (*p*_*Fi×Mj*_) of a cross between females *i* and males *j* is:

$$p_{F_{i}\times M_{j}}\left(t\right)=\frac{f_{F_{i}}\left(t\right)f_{M_{j}}\left(t\right)\varphi_{F_{i}\to M_{j}}}{\sum_{k=1}^{n_{F}}f_{F_{k}}\left(t\right)\sum_{l=1}^{n_{M}}\left(f_{M_{l}}\left(t\right)\varphi_{F_{i}\to M_{l}}\right)}$$

This probability also represents the proportion of the particular offspring genomotype arising from crosses between females *i* and males *j*, assuming that: a) the encounter probability of a pair of genomotypes is only a function of their frequencies in the population, not being affected by other factors such as differential spatial segregation, search rates and conspicuity, among others; b) males are not choosy, meaning they breed indifferently with any female genomotype; c) individual females have similar reproductive successes per reproductive season, regardless of genomotype; d) male genomotypes have similar reproductive capabilities, being equally able to fertilize oocytes (e.g. same sperm quality); and e) the viability and survival of the offspring produced is similar for all cross types. Note that our model aims at assessing how mate choice shapes population dynamics and does not address whether the simulated preferences are adaptive.

We inferred mate preferences (*φ*_*Fi→Mj*_) for each female genomotype from the reproductive dynamics of each population type, that is, triploid- and tetraploid-dominated populations. Thus, we assumed assortative mating to occur only among the self-sustainable PPAA genomotype and allowed PAA females to have a more flexible mate choice pattern, due to their obligatory reproductive interdependency. Although there might be up to four male genomotypes in triploid-dominated populations (Fig 1A), we grouped males according to their functional role in their reproductive dynamics because some males, namely PA and PPAA, produce the same sperm type and, consequently, father the same offspring. We considered three male groups: a) type I males, comprising PA and PPAA males, which produce PA sperm and father PAA offspring with PAA females; b) type II males, the ones from the sympatric *S*. *pyrenaicus* bisexual species (PP genome, P sperm), which father PA offspring with PAA females; and c) type III males, the PAA ones, which produce PAA sperm and father PAAA offspring with PAA females. In triploid-dominated populations, PAA is the most frequent genomotype, followed by the PA and, lastly, by the PAAA one (absent in most populations). These relative frequencies suggest that the male genomotypes which father PAA offspring (PA and PPAA males) may have a higher reproductive success than the ones fathering PA offspring (PP males) and a much higher than the ones fathering PAAA offspring (PAA males), being thus denominated here as type I, type II and type III males, respectively. These differential male reproductive successes may be due to a higher preference of PAA females towards type I males than towards the type II ones (0<*φ*_*FPAA→MII*_ *< φ*_*FPAA→MI*_ *<1*). Nonetheless, we simulated the entire range of preferences towards these two male types (0.002 steps), allowing either type I or type II males to be favored by PAA females (Table 1). We assumed that females reject type III males (*φ*_*FPAA→MIII*_ = 0; Table 1) because the offspring produced from crosses between PAA males and females (PAAA genomotype) is absent in the vast majority of natural populations and, when present, occurs at extremely low frequencies (~1:500).

**Table 1:** Preferences of PAA and PPAA females towards type I, type II and type III males simulated in the model. The preferences of PAA females consist in a flexible mate choice pattern because it includes a certain degree of preference towards both type I and type II males, whereas PPAA females only favor type I males for mating. Note that PPAA females also produce offspring of their own genomotype in crosses with PA males (see text for further details).

	Type I males	Type II males	Type III males	
**Females**	PA and PPAA	PP	PAA	**Mate choice pattern**
**PAA**	0<*φ*_*FPAA→MI*_<1	*φ*_*FPAA→MII*_ = 1-*φ*_*FPAA→MI*_	*φ*_*FPAA→MIII*_ = 0	Flexible
**PPAA**	*φ*_*FPPAA→MI*_ = 1	*φ*_*FPPAA→MII*_ = 0	*φ*_*FPPAA→MIII*_ = 0	Assortative mating

We assessed multiple initial population compositions by varying the relative proportions of each genomotype, namely by increasing or decreasing the frequency of PAA and PPAA genomotypes (Table 2) and, thus, simulating triploid- and tetraploid-dominated populations. For each initial population composition, we ran our model until genomotype frequencies reached stability. Offspring composition at each generation *t* was calculated using the genomotype frequencies at *t*-1 and used as the new parental composition for the next generation (*t*+1). Offspring sex ratios (*R*_*g*_) applied in the model for each genomotype were based on joint data from well-studied triploid- and tetraploid-dominated populations (*R*_*FPA*_ = 0.00, *R*_*MPA*_ = 1.00; *R*_*FPAA*_ = 0.85, *R*_*MPAA*_ = 0.15; *R*_*FPPAA*_ = 0.50, *R*_*FPPAA*_ = 0.50). Note that the sympatric *S*. *pyrenaicus* (PP), whose males also participate in *S*. *alburnoides* reproductive dynamics (Fig 1A), does not belong to the hybrid complex itself. It is an autonomous non-hybrid species with even sex ratios (*R*_*FPP*_ = 0.50, *R*_*MPP*_ = 0.50) and independent population dynamics. For this reason, its frequency among the overall fish population (*S*. *alburnoides* plus *S*. *pyrenaicus*) was kept fixed over all generations of each simulation. The entire range of possible PP frequencies (0<*f*_*PP*_<1, 0.002 steps) was tested in the model.

**Table 2:** Ranges of genomotype frequencies used to generate the initial population compositions for the model. All initial genomotype compositions were aimed at recreating triploid- and tetraploid-dominated populations and used as starting points for all sets of simulations.

Population initial composition	Genomotype frequencies (*f*_*PA*_+*f*_*PAA*_+*f*_*PPAA*_ = 1.0)		
	PA	PAA	PPAA
**Triploid-dominated**	0.0<*f*_*PA*_<0.5	0.5<*f*_*PAA*_<1.0	0.0<*f*_*PPAA*_<0.5
**Tetraploid-dominated**	0.0<*f*_*PA*_<0.5	0.0<*f*_*PAA*_<0.5	0.5<*f*_*PPAA*_<1.0

Thus, the overall dynamics of our theoretical model is

$$f_{g}\left(t+1\right)=R_{g}\sum_{i}\sum_{j}\left(p_{F_{i}\times M_{j}}\left(t\right)b_{g}\left(F_{i}\times M_{j}\right)\right)$$

where *b*_*g*_ is a binary variable assuming a value of 1 when the cross between females *i* and males *j* originates offspring of genomotype *g* and a value of 0 otherwise. All simulations were performed in R software v2.15.2 [23].

### Mate Choice Trials

We assessed mate preferences of PAA females through mate choice experiments. Trial females were allowed to choose among the male genomotypes present in a triploid-dominated population (Tagus drainage). This population was also used as one of the main references for the model, namely regarding sex-ratios. A random sample (*N* = 41) of *S*. *alburnoides* (*N* = 25: *f*_*MPA*_ = 0.28, *f*_*FPAA*_ = 0.56, *f*_*MPAA*_ = 0.12, *f*_*MPPAA*_ = 0.04) and *S*. *pyrenaicus* (*N* = 16: *f*_*FPP*_ = 0.50, *f*_*MPP*_ = 0.50) was collected from Ocreza River during the reproductive season. The capture was performed randomly, trying to cover all available habitats in order to guarantee that the genomotype composition of the sample would be representative of the one found in the studied population. Note that *S*. *alburnoides* and *S*. *pyrenaicus* are threatened fishes, classified as Vulnerable and Endangered [24], respectively, which raises ethical challenges to the capture of larger samples. The individuals were sexed by applying a mild and brief pressure on the abdomen, forcing the extrusion of a few gametes. The fish were transported to the laboratory and small fin clips were used to assess the genomotype of each individual by flow cytometry [25] and sequencing of the *β-actin* gene [26]. Individuals were kept together in a maintenance tank (250 L) with a 14h/10h light/dark cycle, mimicking the natural conditions of the reproductive season, and were fed twice a day with an adequate amount of frozen bloodworms and brine shrimp. The water quality was assessed on a weekly basis.

**Fig 3:**
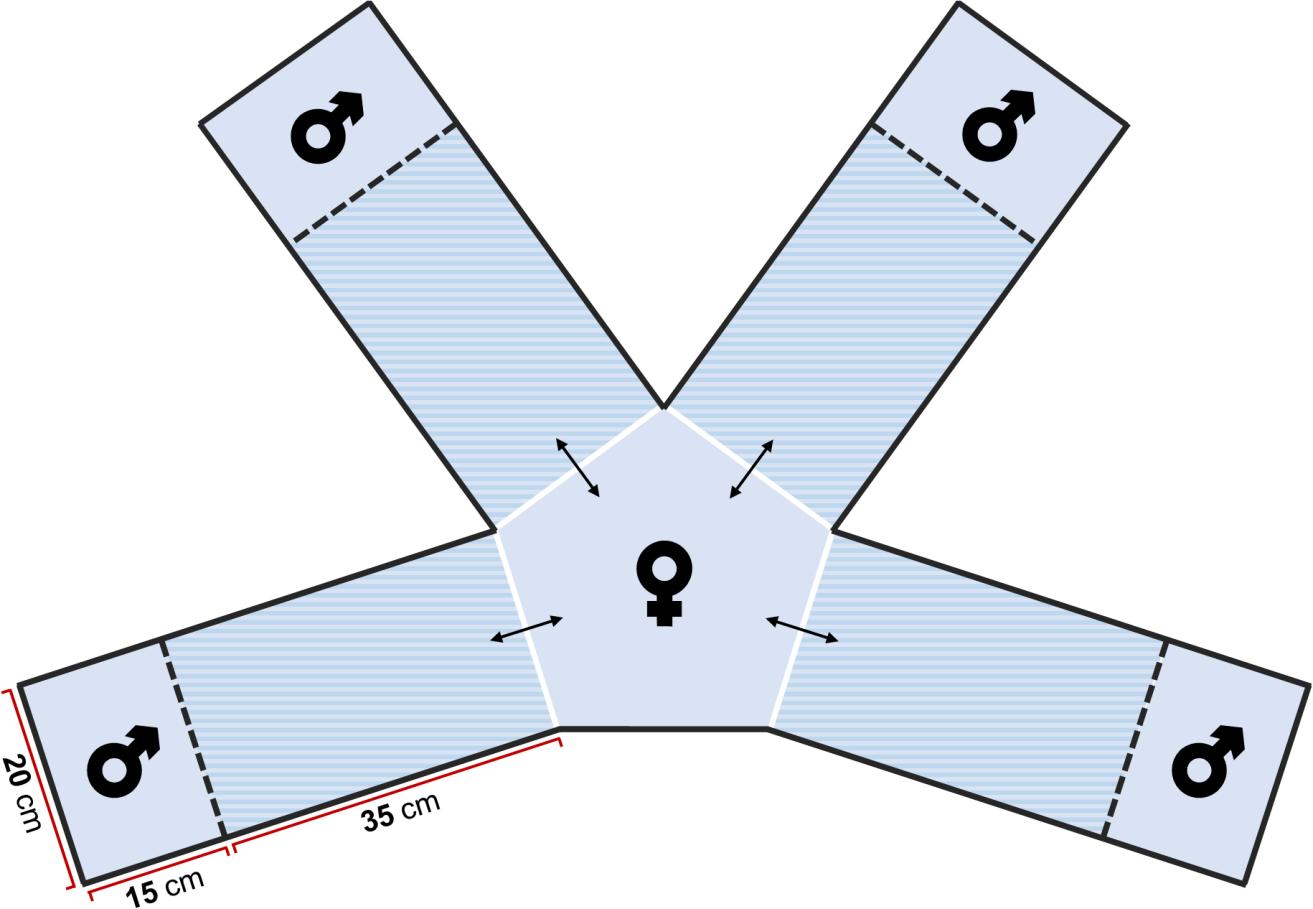
Experimental tank specially designed for the study of *S*. *alburnoides* mate choice. The choice areas for each male genomotype are dashed. The proportion of time spent by females in each of these areas was used as a measure of preference, being the central area considered neutral. Male compartments were delimited by transparent perforated acrylic plates, allowing the passage of all types of stimuli between male and female.

The experimental trials started after a two-week habituation period to captivity, also ensuring that the small portion of tissue collected from the terminal edge of the fins was fully regrown. Individual recognition was performed using scale patterns [27]. Each experimental trial was conducted in a mate choice tank, specially designed for the purpose (Fig 3). In each trial (*N* = 11), a single individual of each male genomotype (PA, PAA, PPAA and PP) was inserted randomly in each of the male compartments and a single PAA female in the central neutral area of the experimental tank. Due to the rarity of some male genomotypes, some stimulus males were used more than once in the affiliation trials (contrary to females, which were never repeated). Note that adult genomotypes have distinct characteristic lengths [28, 29], making it impossible to isolate the effect of fish size (mean standard lengths: ♂PA, 5.38 cm; ♀PAA, 5.84 cm; ♂PAA, 7.20 cm; ♂PPAA, 5.40 cm; ♂PP, 7.09 cm). Trial females (*N* = 11) were allowed to swim freely across the tank and visit each of the males during a period of 1h 30min. The trials were recorded using a digital camera for ulterior video analysis in which the proportion of time spent by females near each male was measured. The first half hour of each trial was considered habituation period to the experimental tank and, thus, discarded from the analyses. The proportion of time spent by females near each male was used as a measure of preference [30] and compared among male genomotypes (PA, PAA, PPAA and PP) and groups (type I, type II and type III) using repeated measures ANOVA. Normality and sphericity assumptions were tested with Shapiro-Wilk’s and Mauchly’s tests, respectively. When needed, all frequencies were transformed using the arcsine of the square root in order to achieve normality. Repeated measures ANOVA is quite robust dealing with normality violations, thus, slight deviations were considered acceptable. When our data violated the sphericity assumption, a Greenhouse-Geisser correction was used. Post-hoc tests were carried out using a Bonferroni correction for multiple comparisons. Lastly, the genomotype composition of the sample collected and the female preferences obtained from the mate choice trials, particularly the frequency of the sympatric *S*. *pyrenaicus* (*f*_*PP*_) and the preference of PAA females towards type I males (*φ*_*FPAA→MI*_), were used to run the model. All statistical procedures were performed in StatSoft Statistica v12 [31].

## Results

**Fig 4:**
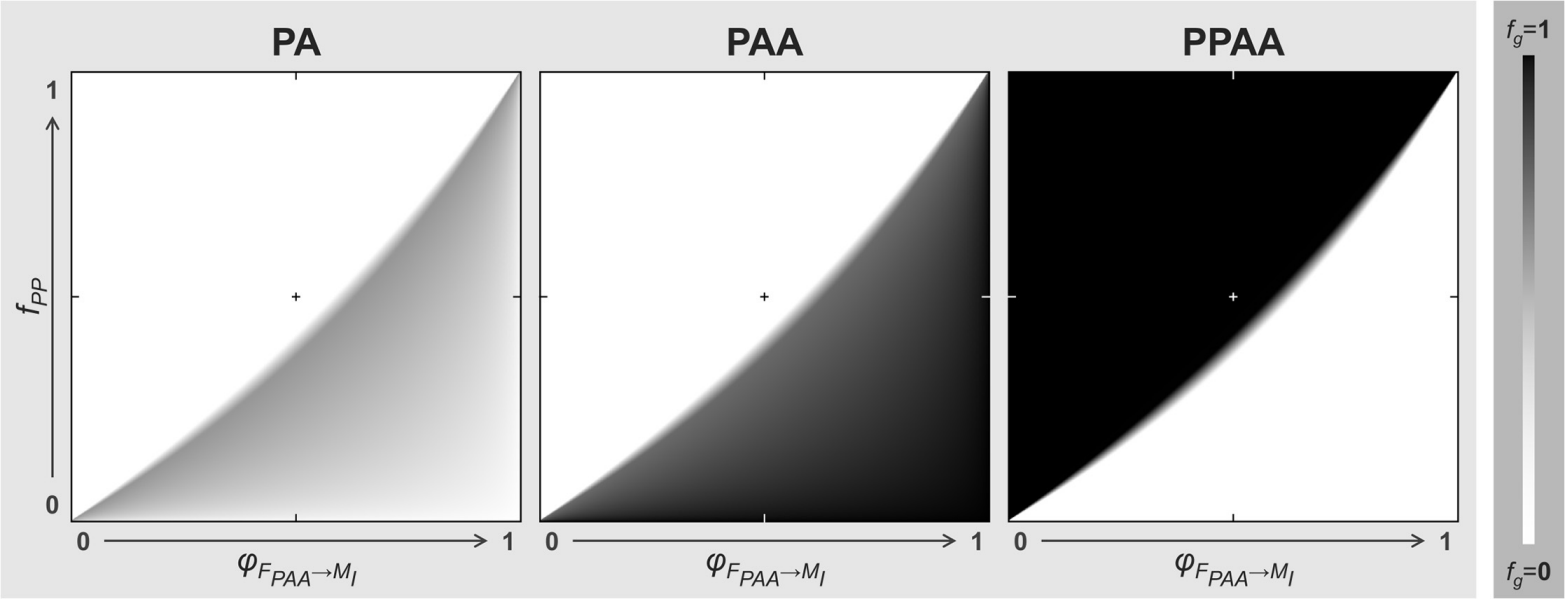
Genomotype frequencies at the equilibria predicted by the model. Relative frequencies of the three *S*. *alburnoides* genomotypes (PA, PAA and PPAA) range from *f*_*g*_ = 0 (white) to *f*_*g*_ = 1 (black) as a function of PAA females’ preference towards type I males (*φ*_*FPAA→MI*_; Table 1) and of the frequency of the sympatric *S*. *pyrenaicus* non-hybrid species (*f*_*PP*_). Note that, although a 0<*f*_*PP*_<1 range is shown on the *y*-axis, the model cannot operate on *f*_*PP*_ = 1 because it represents a population exclusively constituted by *S*. *pyrenaicus* specimens, in which the *S*. *alburnoides* ones are absent.

For all initial genomotype compositions (see Table 2), we simulated 500 distinct PP frequencies (0<*fPP*<1, 0.002 steps) and 500 distinct mate preferences of PAA females towards type I males (0<*φ*_*FPAA→MI*_<1, 0.002 steps; Table 1), totalizing 250 000 distinct scenarios per initial genomotype composition. Equilibrium was reached for both triploid- and tetraploid-dominated populations under multiple scenarios. No relation was found between the initial genomotype composition and the evolutionary pathway followed by simulated populations because the equilibrium reached was similar for any initial composition. However, the two parameters we studied, namely, mate preferences and frequency of the sympatric *S*. *pyrenaicus*, influenced the final equilibrium (Fig 4). Most scenarios that favored tetraploidization led populations to be exclusively composed by the self-sustainable PPAA genomotype (*f*_*PPAA*_ = 1.0), whereas the ones favoring the dominance of the PAA genomotype also allowed the maintenance of the PA one (Fig 4), derived from the PAA females’ variable preference degree towards type II males (PP genome, P sperm). In general, these theoretical genomotype compositions do not differ significantly from the ones observed in natural populations (Fig 1B).

**Fig 5:**
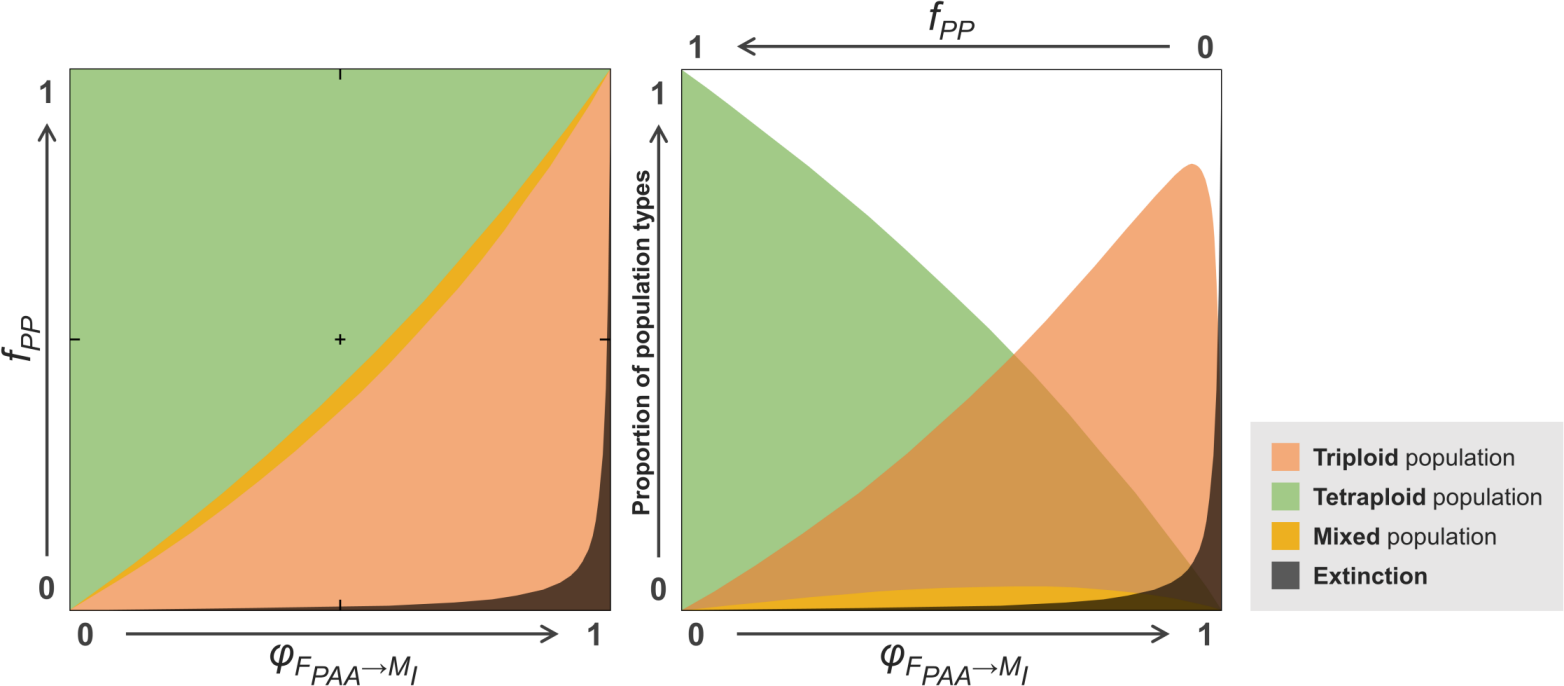
Range of scenarios leading to all population types predicted by the model. Tetraploid-dominated populations exclusively constituted by the PPAA genomotype are represented in green, whereas triploid-dominated populations composed by the PAA and the PA genomotypes are shown in red. Yellow represents the narrow area of scenarios leading to stable populations comprising the three genomotypes (PA, PAA and PPAA) in equilibrium. Lastly, the dark area represents populations exclusively constituted by the interdependent PAA genomotype and, therefore, the respective set of scenarios was considered to lead populations to extinction (see text for more details).

The overall outcome of our theoretical model shows that the persistence of triploid- and tetraploid-dominated populations are favored by opposite forces, although a narrow range of scenarios allowed the co-existence of both triploid and tetraploid genomotypes in the same population (Fig 5). Whereas lower PP frequencies and stronger preferences of PAA females towards type I males (PA and PPAA) seem to favor the persistence of triploid-dominated populations, higher PP frequencies and stronger preferences of PAA females towards type II males (PP) seem to route populations towards tetraploidization (Fig 5). From all 250 000 simulated scenarios, 55.8% led to tetraploid-dominated populations, 38.8% stabilized in triploid-dominated ones, 2.9% allowed the equilibrated coexistence of the PAA and PPAA genomotypes, and 2.5% led populations to be exclusively composed by the PAA genomotype (Fig 5). Note that populations only constituted by the PAA genomotype are not viable because *S*. *alburnoides* triploid-dominated populations cannot persist without genomotype variability (see Introduction; Fig 1A). Thus, this last outcome was considered to represent extinction.

**Fig 6:**
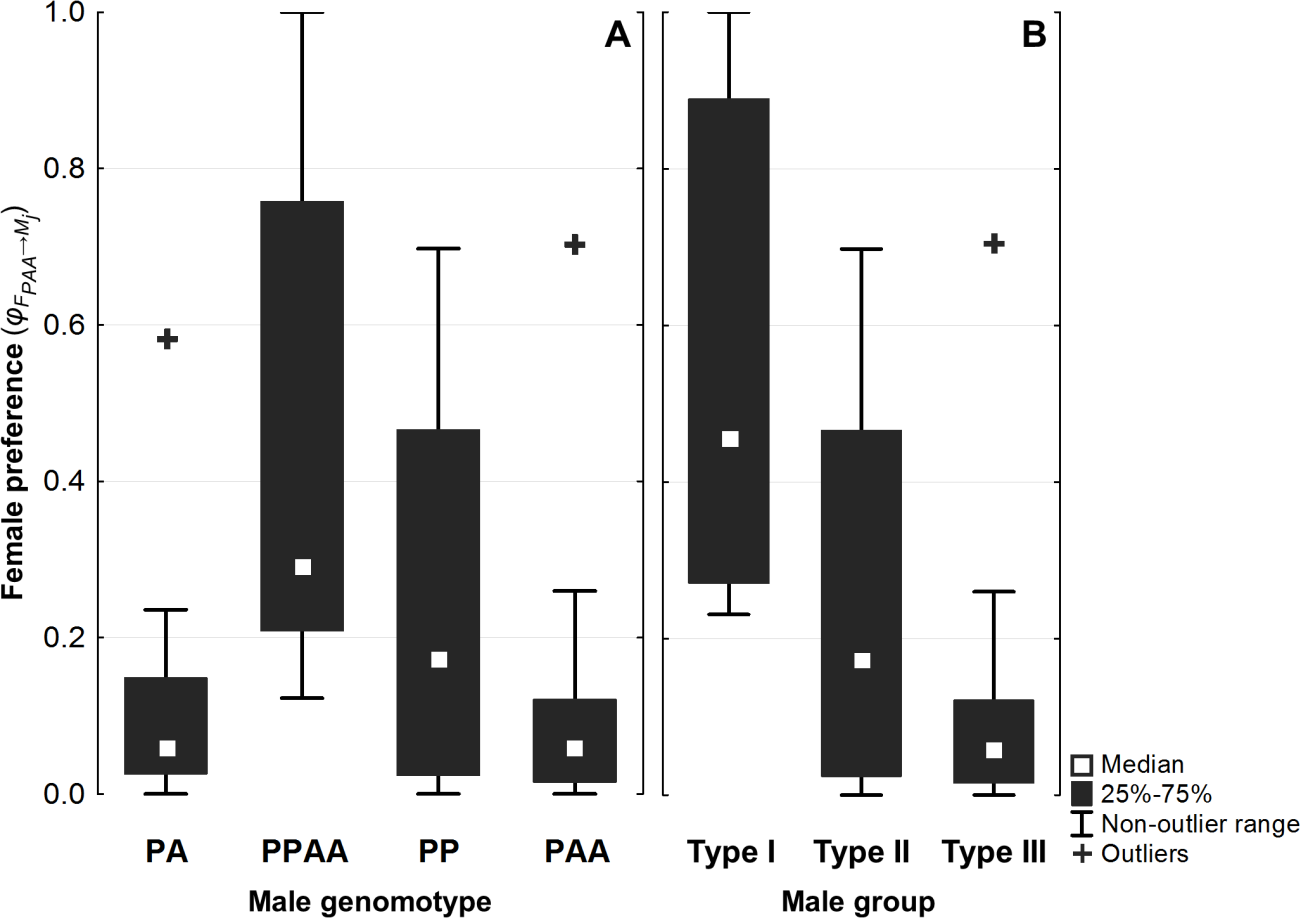
Mate choice results. Proportion of time spent by tested PAA females (*N* = 11) near each male genomotype (**A**: PA, PPAA, PP and PAA) and by male group (**B**: type I, type II and type III). These results obtained from the affiliation trials were used as measures of female preference (means: *φ*_*FPAA→MPA*_ = 0.13, *φ*_*FPAA→MPPAA*_ = 0.45, *φ*_*FPAA→MPP*_ = 0.26, *φ*_*FPAA→MPAA*_ = 0.13; *φ*_*FPAA→MI*_ = 0.58, *φ*_*FPAA→MII*_ = 0.26, *φ*_*FPAA→MIII*_ = 0.13).

In order to experimentally evaluate the role of female preferences (one of the main parameters of our model) in *S*. *alburnoides* reproductive dynamics, we assessed the mate preferences of PAA females in affiliation trials (Fig 6A; S1 Table). Repeated measures ANOVA revealed that tested PAA females (*N* = 11) showed differential mate preferences towards the available male genomotypes (i.e. PA, PAA, PPAA and PP males) (*F*_3,30_ = 3.834, *p* = 0.019). Post-hoc tests using the Bonferroni correction revealed that PAA females had a significant higher preference for PPAA males than for PA (*p* = 0.041) and PAA males (*p* = 0.035). Males from the sympatric *S*. *pyrenaicus* non-hybrid species (PP) were in an intermediate position, not differing significantly from any of the *S*. *alburnoides* male genomotypes. Note that, as previously stated, adult genomotypes have distinct typical lengths [28, 29], which does not allow to exclude the effect of fish size from our results. However, fish size seems unlikely to play a major role because females showed distinct affiliation tendencies towards male genomotypes with similar average standard lengths (PA vs. PPAA; PP vs. PAA; Fig 6A).

In order to compare the experimental results with the outcome our model, the set of preference levels obtained from the mate choice trials was reorganized by male type, namely type I (PA and PPAA), type II (PP) and type III males (PAA). Thus, PAA females’ preference levels towards PA and PPAA male genomotypes (type I males) were summed for each tested female, reorganizing the data according to the offspring genomotype females would produce with each male group (PAA, PA and PAAA with type I, type II and type III males, respectively). All statistical analyses were repeated for this new male structure (Fig 6B). Repeated measures ANOVA revealed that PAA females showed a differential affiliation tendency towards the three male groups (*F*_2,20_ = 6.597, *p* = 0.006). Post-hoc tests using the Bonferroni correction revealed that PAA females’ preference towards type I males were significantly higher than towards type III males (*p* = 0.007) and nearly significantly higher than towards type II males (*p* = 0.052). However, their affiliation tendency was similar between type II and type III males.

Lastly, the frequency of the sympatric *S*. *pyrenaicus* species observed in the studied natural population (*f*_*PP*_ = 0.39) and the average joint preference of PAA females towards type I males (PA and PPAA) obtained from the mate choice trials (*φ*_*FPAA→MI*_ = 0.58) were used to run the theoretical model. Simulated *S*. *alburnoides* genomotype frequencies (*f*_*PA*_ = 0.38, *f*_*PAA*_ = 0.62, *f*_*PPAA*_ = 0.00) were close to the ones observed in the referred natural population (*f*_*PA*_ = 0.28, *f*_*PAA*_ = 0.68, *f*_*PPAA*_ = 0.04).

## Discussion

The irrelevant role of the initial *S*. *alburnoides* genomotype composition over the final equilibrium reached in each simulation reveals that even a low frequency of the PPAA genomotype in a triploid-dominated population may tetraploidize it and even a low frequency of the PAA genomotype in a tetraploid-dominated population may triploidize it. Thus, the evolutionary route a given population will follow is independent of its current genomotype composition, but, according to our findings, seems highly influenced by female preferences and frequency of the sympatric *Squalius* species. A closer look at the role of both these forces in shaping *S*. *alburnoides* genomotype composition over time reveals that their relevance relies on the production of PA males. This intermediary genomotype seems to play a central role in tetraploidizing populations, but it is also indispensable for the persistence of triploid-dominated populations. This happens because both PAA and PPAA females restock their own genomotype by crossing with PA males. On the one hand, when the frequency of the sympatric *S*. *pyrenaicus* (PP) is high and/or when PAA females show a higher preference towards these males (conditions that favor tetraploidization; Fig 5), crosses between PAA females (A oocytes) and PP males (P sperm) become more frequent, increasing the frequency of the PA genomotype in the population. In turn, crosses between PPAA females (PA oocytes) and PA males (PA sperm) become more frequent, leading to an overall increase of the PPAA genomotype and, consequently, tetraploidizing the population. On the other hand, when the sympatric *S*. *pyrenaicus* non-hybrid species (PP) is less frequent and/or the PAA females’ preference towards type I males (PA and PPAA) is higher than it is towards the type II ones (PP) (conditions that favor triploidization; Fig 5), crosses between PAA females (A oocytes) and PA males (PA sperm) become more frequent, leading to an overall increase of the PAA genomotype and, consequently, triploidizing the population. Although this last scenario seems to lead to a struggle between the dominance of PAA and PPAA genomotypes (triploidization vs. tetraploidization), it actually leads to the persistence of triploid-dominated populations. Although, at first sight, this outcome may look unexpected due to the fact that PAA females, contrary to the PPAA ones, need an intermediary step (PA production; Fig 1A) in order to restock their own genomotype, the explanation lies on the characteristic female-biased sex ratio of the PAA genomotype. Its higher proportion of females (~85%) represents an advantage that compensates the assortative mating occurring among the PPAA genomotype and hinders tetraploidization in some scenarios (Fig 5).

If the stability of triploid- and tetraploid-dominated populations depended exclusively on the two factors assessed by our theoretical model (frequency of the sympatric *Squalius* bisexual species and mate choice pattern of PAA females) and if the observed values of both these variables were random among natural populations, the overall outcome of our model indicates that 55.8% of *S*. *alburnoides* natural populations would be tetraploid-dominated (against 38.8% of triploid-dominated ones), because the set of conditions favoring tetraploidization is wider than the one leading to triploidization. However, triploid-dominated populations abound across *S*. *alburnoides* geographic range and only two tetraploid-dominated populations were found so far, suggesting that the values composing the range of the studied factors do not seem to be equally probable to occur among natural populations. Both known tetraploid-dominated populations [14] occur in northern Portugal, a geographic area where the sympatric *Squalius* species, *S*. *carolitertii* (CC genome), has a Least Concern status, being more common and abundant than the sympatric species of southern regions, *S*. *pyrenaicus* and *S*. *aradensis*, classified respectively as Endangered and Critically Endangered [24]. However, although this higher frequency of the bisexual *Squalius* species might have helped the tetraploidization of those tetraploid-dominated populations (composed almost exclusively by CCAA males and females), most populations of the northern region are also triploid-dominated (CAA genomotype). In these other populations, the preference of triploid females towards type I males is probably high enough to promote triploidization and counter the effect of the assortative mating occurring among the tetraploid genomotype (Fig 5). Note that, although the genomotypes of the northern region include C and not P genome, their reproductive modes are the same and, thus, the reproductive dynamics of those populations is similar to the one shown in Fig 1.

The results of the experimental mate choice trials were in agreement with the dominance of triploid populations in nature because PAA females showed a higher affiliation tendency with type I males (PA and PPAA), crosses that produce PAA offspring, thus, contributing to triploidize populations. However, female preference differed significantly between the two type I male genomotypes, with PPAA being favored over PA. This difference may be related to the fact that PPAA males undergo meiosis as reproductive mode and, therefore, contribute to a higher genetic variability of the offspring, a factor already proposed as relevant when choosing a mate [32–34]. The same does not apply to the PA males because they produce their gametes through clonal gametogenesis and, thus, the genetic variability of their offspring only comes from the mother. Future additional mate choice trials in a triploid-dominated population in which the PPAA genomotype is absent may be useful to understand the observed difference, in order to assess if the preference of PAA females towards PA males is higher when no PPAA males are available.

Type III males (PAA) were the least preferred choice of triploid females, a predictable result considering the extreme rarity of the PAAA genomotype (the offspring produced from crosses between PAA males and females) in natural populations. However, this result is still particularly interesting because PAA females seem to avoid mating with males of their own genomotype (disassortative mate choice) in order to produce offspring of their own genomotype (assortative mate choice). To our knowledge, this assortative-disassortative mate choice pattern was never reported before.

Because hybrid organisms with nonsexual reproductive modes have altered gametogenesis and lack regular sexual mechanisms (i.e. normal amphimixis), they were for long considered evolutionary dead-ends [35]. However, several studies over the last decades have shown otherwise [16, 36]. Actually, the intricate reproductive dynamics of most hybrid complexes allow a multiplicity of alternative evolutionary pathways along which organisms may evolve. Our results suggest a key role for mate choice in driving such pathways. Although assortative mating may favor tetraploidization and route populations towards hybrid speciation, the other mate choice patterns occurring among hybrids seem able to counter its effect and maintain populations in its triploid-dominated state. Nonetheless, the role of the bisexual *Squalius* species in *S*. *alburnoides* reproductive dynamics seems to be equally relevant in routing populations towards tetraploidization. Hybrid speciation seems only possible if the sympatric parental species is frequent in the population, suggesting that sympatry is mandatory for speciation in this hybrid complex, contrary to what is commonly argued [37–40]. Indeed, parental bisexual species may play a persistent key role in hybrid systems, an effect that can ultimately influence mate choice [30, 41].

Our findings add an important and almost neglected piece to the puzzling persistence of some hybrid animal populations without speciation or extinction. Among vertebrates, namely in amphibians and fishes [16, 40, 42–46], several successful hybrid populations have been reported over the years, some with independent reproductive dynamics, but, to our knowledge, this is the first assessment on the influence of mate choice in routing the evolutionary pathways of such organisms, bridging theoretical and experimental approaches. The role of mate choice uncovered in our study may be applicable to other similar hybrid systems, that is, hybrid populations upheld by sexual and nonsexual reproductive modes.

## Supporting Information

S1 Table Data obtained from mate choice trials. Preference values refer to the proportion of time tested females (PAA, *N* = 11) spent interacting with each male genomotype.
(DOCX)
