# Supplementary material for: Mate Choice Drives Evolutionary Stability in a Hybrid Complex
Source: PLoS One. 2015 Jul 16;10(7):e0132760. doi: 10.1371/journal.pone.0132760 (PMC4504517; doi:10.1371/journal.pone.0132760)
Supplement: S1 Table — Preference values refer to the proportion of time tested females (PAA, N = 11) spent interacting with each male genomotype. (DOCX) [file pone.0132760.s001.docx]

| **Females** | **Preference** | | | |
| --- | --- | --- | --- | --- |
| (PAA) | **PA ♂** | **PAA ♂** | **PPAA ♂** | **PP ♂** |
| #1 | 0.060 | 0.705 | 0.171 | 0.036 |
| #2 | 0.000 | 0.000 | 1.000 | 0.000 |
| #3 | 0.583 | 0.038 | 0.292 | 0.078 |
| #4 | 0.039 | 0.016 | 0.231 | 0.698 |
| #5 | 0.148 | 0.260 | 0.123 | 0.360 |
| #6 | 0.132 | 0.069 | 0.757 | 0.024 |
| #7 | 0.125 | 0.083 | 0.533 | 0.173 |
| #8 | 0.236 | 0.121 | 0.209 | 0.378 |
| #9 | 0.035 | 0.052 | 0.291 | 0.593 |
| #10 | 0.026 | 0.059 | 0.429 | 0.466 |
| #11 | 0.024 | 0.009 | 0.949 | 0.009 |
